# In-situ ToF-SIMS analyses of deuterium re-distribution in austenitic steel AISI 304L under mechanical load

**DOI:** 10.1038/s41598-020-60370-2

**Published:** 2020-02-27

**Authors:** Andreas Röhsler, Oded Sobol, Hannu Hänninen, Thomas Böllinghaus

**Affiliations:** 1 Federal Institute for Materials Research and Testing, Department for Component Safety, Berlin, 12205 Germany; 2 Aalto University, Department of Mechanical Engineering, Espoo, 00076 Finland

**Keywords:** Structural materials, Techniques and instrumentation, Engineering

## Abstract

Hydrocarbons fuel our economy. Furthermore, intermediate goods and consumer products are often hydrocarbon-based. Beside all the progress they made possible, hydrogen-containing substances can have severe detrimental effects on materials exposed to them. Hydrogen-assisted failure of iron alloys has been recognised more than a century ago. The present study aims to providing further insight into the degradation of the austenitic stainless steel AISI 304L (EN 1.4307) exposed to hydrogen. To this end, samples were electrochemically charged with the hydrogen isotope deuterium (^2^H, D) and analysed by scanning electron microscopy (SEM), electron back-scatter diffraction (EBSD) and time-of-flight secondary ion mass spectrometry (ToF-SIMS). It was found that deuterium caused a phase transformation from the original *γ* austenite into *ε*- and *α*’-martensite. Despite their low solubility for hydrogen, viz. deuterium, the newly formed phases showed high deuterium concentration which was attributed to the increased density of traps. Information about the behaviour of deuterium in the material subjected to external mechanical load was gathered. A four-point-bending device was developed for this purpose. This allowed to analyse *in-situ* pre-charged samples in the ToF-SIMS during the application of external mechanical load. The results indicate a movement of deuterium towards the regions of highest stress.

## Introduction

Researchers already warned against climate change in the 1970s, but only the “special report on the impacts of global warming of 1.5 ° C above pre-industrial levels”, published by the Intergovernmental Panel on Climate Change (IPCC) together with massive civic engagement, brought this topic back to the agendas of policymakers^1,2^. Man-made global warming ultimately causes extinction of species, desertification, poverty and migration and its ramifications will affect mankind as a whole. Taking action to reduce emissions of greenhouse gases in sectors such as energy production, transportation, mobility and industry are now more important than ever.

Among other measures, the transition towards renewable energy sources like wind and solar power is necessary and inevitable to mitigate the emission of greenhouse gases. However, these technologies face us with the problem of storing the generated electricity. Converting electrical energy into chemical energy through the formation of hydrogen and methane is a suitable way for this purpose. These gases can be stored, transported and used to re-generate electrical energy. Establishing a reliable infrastructure for this power-to-gas grid requires further investigation in every aspect of the chain, from conversion efficiency to safety of components such as pipelines exposed to high concentrations of hydrogen. This is necessary because the suitability of materials used for this hydrogen-infrastructure is still not fully clarified^3^. Moreover, hydrogen-induced degradation of such components can cause high economic and environmental costs^4^.

Hydrogen-assisted damages of steels already used in the fossil energy infrastructure are a long-known phenomenon with different facets^5–7^. Accelerated fatigue crack growth^8,9^, blistering^10,11^, degradation of strength and ductility^12–14^ and delayed fracture^15,16^ are typical features caused by the entry and accumulation of hydrogen in the material during service. Another effect that is still under debate is the hydrogen-induced transformation of the face-centred cubic (FCC) structure via the close-packed hexagonal (HCP) into the body-centred cubic (BCC) structure^17–20^. During this phase transformation, the properties of the alloy change dramatically. The FCC austenite possesses a high mechanical ductility and high solubility but low diffusivity for hydrogen, whereas the newly formed BCC martensite is more brittle and has a lower solubility and higher diffusivity for hydrogen as compared to its parent lattice^21,22^. These altered diffusion properties facilitate hydrogen uptake that can induce more phase transformation. An unfavourable cycle that accelerates the materials degradation may begin.

External and internal mechanical stress and strain can support the uptake and transport of hydrogen. Not only that hydrogen atoms can be transported by moving dislocations during plastic deformation^23^, an elastically expanded lattice offers faster pathways as well. Hydrogen will then move from regions under compressive strain/stress to the areas under tension. This is referred to as *Gorsky effect*^24^. It is especially remarkable that no external stress is needed for this process to occur. Atomic and molecular hydrogen alone can provide sufficient strain to expand the metallic lattice.

A result of the changes in mechanical properties mentioned above, is a transition of the fracture mode and accelerated cracking. Eventually, the lifetime of structural components exposed to hydrogen is reduced. If the energy transition shall be realised, further research on hydrogen-assisted damages is crucial both to successfully implement renewable energies and to save dwindling resources.

One goal of the present study is to investigate structural and microstructural changes induced by hydrogen. For this purpose, samples made from austenitic stainless steel AISI 304L were electrochemically charged with deuterium and analysed by electron back-scatter diffraction (EBSD) and scanning electron microscopy (SEM). The work further aims to generate more knowledge about both the capability of different phase constituents to bind or trap hydrogen and the mobility of hydrogen in the microstructure under external mechanical load. To this end, the distribution of hydrogen and its behaviour within the specimen before and during the application of mechanical load were examined by time-of-flight-secondary ion mass spectrometry (ToF-SIMS). Due to the difficulties to distinguish between artificially introduced hydrogen and abundant hydrogen in the rest gas of the ToF-SIMS, deuterium (^2^H, D) was used as a tracer for hydrogen (H) .

## Results and Discussion

### Deuterium-induced structural changes

**Figure 1:**
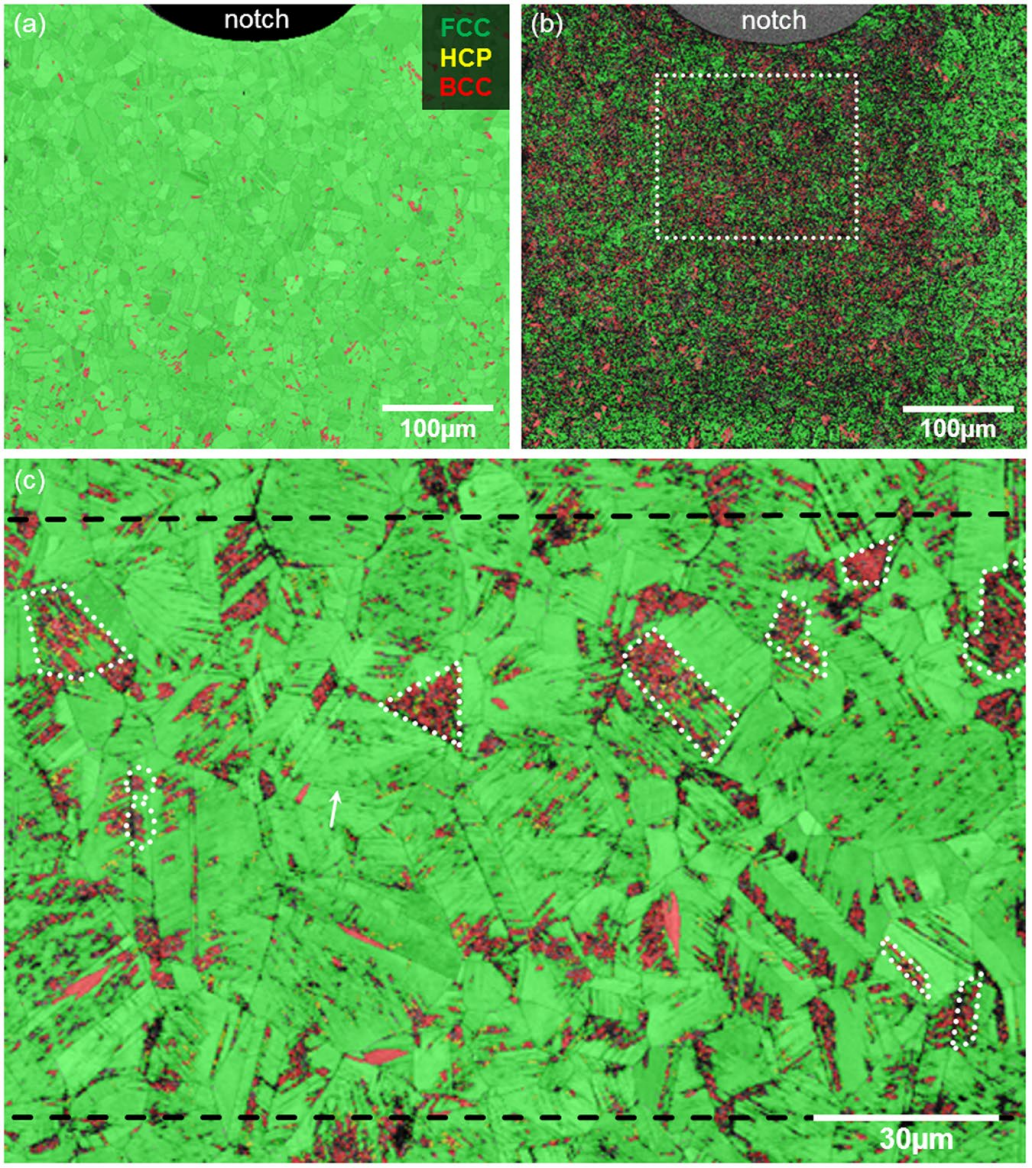
The overlay of pattern quality (PQ) and phase distribution map of the sample *before* charging is shown in (**a**). The same region *after* electrochemical charging for five days and ToF-SIMS experiments is depicted in (**b**). The dashed frame in (**b**) indicates the magnified area within the field of analysis in (**c**). Dotted white lines indicate newly-formed martensite, a white arrow marks slip bands. The dashed black lines represent the same ROI shown in Fig. 3.

**Figure 2:**
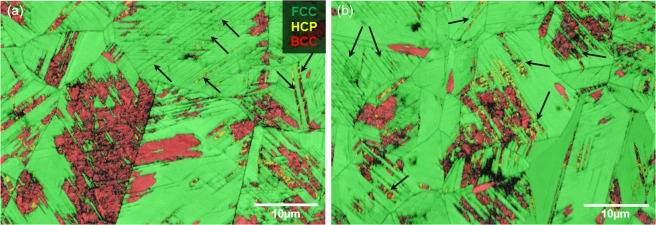
The overlay of pattern quality (PQ) and phase distribution map of the surface *after* electrochemical charging for five days. Both images (**a**,**b**) were taken outside the region sputtered and analysed by ToF-SIMS. Black arrows indicate slip bands as nucleation sites for *ϵ*- and *α*’-martensite.

The results of the EBSD analyses before and after electrochemical charging for five days are shown in Figs. 1 and 2. Figure 1(a) shows the materials condition after sample preparation (cf. section *Materials and preparation*). The surface consisted mainly of austenite, indicated by green colour, with approximately 4% of residual martensite coloured in red. This martensite stems from cold rolling of the sheet material.

After five days of electrochemical charging (cf. Chapter *Electrochemical charging*) and ToF-SIMS analyses, the surface underwent changes. EBSD is able to illustrate the transformation of the original FCC phase into BCC martensite (cf. Fig. 1(b)) by analysing the same region of interest (ROI) as before charging. Due to the impact of the Cs^+^ beam, used for removing contaminants from the surface, the sputtered region is visible. It was damaged more severely and contained a larger fraction of martensite. Areas where no diffraction bands could be indexed, i.e. measurement points with zero solution for the Kikuchi patterns, are indicated in black colour in the phase maps. The high number of these locally unsolved patterns is due to the higher thickness and density of the damaged surface.

The results show that the influence of the sputter beam must not be neglected in the discussion of the ToF-SIMS results. For other methods, such as dynamic SIMS, where the energy and flux of the primary ion beam are much higher, this is particularly important.

 Figure 1(c) shows the pattern quality and phase maps that are marked by the white dashed square in Fig. 1(b). It has a higher magnification and was taken with a smaller step size of the rastering electron beam. This significantly improved the image quality and its information content. A reduced step size thus leads to slower but more precise measurements. This is why the non-indexed measurement points in (b) are reduced dramatically. Figure 1(c), thus, shows that the influence of the sputter beam is overestimated in the phase distribution map in (b). Furthermore, Fig. 1(c) not only displays the BCC *α*’-martensite coloured in red but also the yellow-hued *ϵ*-martensite. As described in the literature, this hexagonal phase reflects the intermediate step in the transformation sequence of the FCC austenite into BCC martensite^25–28^.

To fully proof that the phase transformation was not induced by sputtering in the ToF-SIMS, but by deuterium-charging, EBSD images were taken outside of the sputter crater. Figure 2(a,b) exemplary show such locations. The ingress and accumulation of deuterium generated *γ* → *ϵ* → *α*’- transformation on slip bands. The formation of martensite on such deformation bands during quenching and straining was already described by Olson and Cohen^29^. Analogous to their description, stacking faults (SF) formed due to high localised mechanical strain that was induced by deuterium accumulation. These SFs were the basis of both *ϵ*-martensite and mechanical twins. Stacking faults arranged on every second {111}-plane of the FCC lattice, lead to an ABABAB stacking sequence and thereafter *ϵ*-martensite^30^. Within the newly formed *ϵ*-regions, acting as nucleation sites, *α*’-martensite evolved by a further shear of atomic planes due to the ingress of more deuterium into the lattice. Black arrows in Fig. 2 indicate slip bands as nucleation sites for phase transformation.

Meta-stable austenitic steels with a stacking fault energy (SFE) below 18-20 mJ/m^2^ tend to form stacking faults when subjected to mechanical load^31^. The calculated SFE of the investigated alloy AISI 304L is, depending on the equation applied, between 15.7 and 18.1 mJ/m^2^ ^32^. Deuterium-induced *γ* → *ϵ* → *α*′- transformation was thus expected.

### Distribution of deuterium in the material

**Figure 3:**
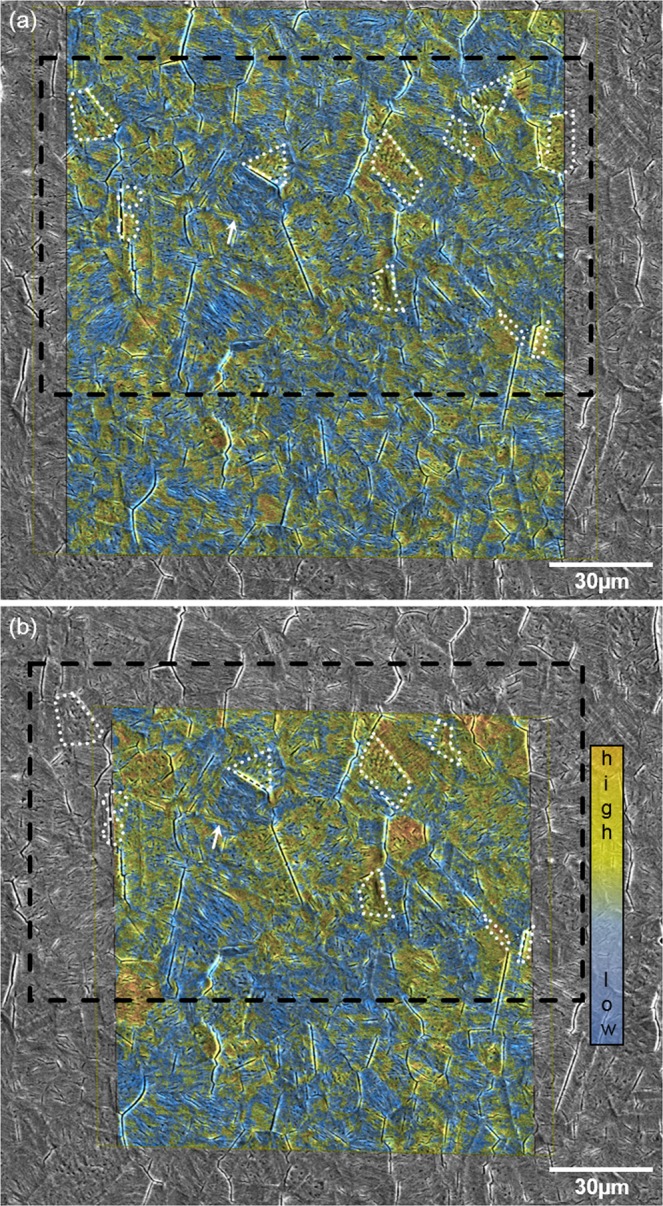
The overlay of an SEM micrograph and the SIMS data after principal component analysis (PCA) *before* (**a**) and *during* applying mechanical load (**b**). The distribution of deuterium and its covariant fragments is reflected as principal component two (PC2) in (**a**) and PC1 in (**b**). Brighter colours refer to a higher intensity of the signal. For a better orientation, the same ROI as shown in Fig. 1(c) is marked by dashed black lines, whereas the same features are marked by dotted white lines. Due to the sample drift during bending and temperature variations, the image in (**b**) had to be cropped. The notch of the sample is located appr. 85 μm above the SIMS images.

The distribution of deuterium, used as a tracer for hydrogen, and its behaviour under mechanical load was investigated by in-situ ToF-SIMS experiments. Figure 3 presents the fusion of a SEM image and the SIMS data of the same region. Here the distribution of deuterium is represented by bright colour, whereas blue indicates regions with a low intensity of the D signal.

Severe cracking and roughening of the surface are visible in the SEM micrograph taken after electrochemical charging and ToF-SIMS experiments. This can be attributed to a number of effects: The formation of martensite, which is connected to a shape deformation^33^; the so-called hydrogen-enhanced decohesion of the lattice (HEDE) and alterations in the stress state of the surface after charging^34^. HEDE is a mechanism that describes the weakening and loss of the cohesive strength due to the influence of hydrogen. A hydrogen-induced decrease in the electron-charge density between metal-metal atoms results in the weakening of atomic bonds^35^. The ingress and accumulation of deuterium in the surface of the material expanded the metal lattice. This expansion induced a compressive stress^36^. As soon as the cathodic potential was removed, i.e. electrochemical charging ceased, deuterium rearrangement and effusion began. That way prevailing compressive stresses within the deuterium-saturated layers transformed into tensile stress due to contraction of the lattice. The phenomenon was described e.g. by Mathias *et al*.^34,37^. The mechanical energy introduced to the matrix was partially relieved by surface cracking and martensite formation. However, this internal stress was not relieved completely so that certain regions in the surface remained under tensile stress.

It is evident the above-mentioned mechanism is not solely responsible for crack formation. Djukic *et al*. e.g. reviewed recently the interplay of hydrogen-enhanced localised plasticity (HELP) and the HEDE mechanism in steels^38^. For this reason, it is assumed that surface cracking has developed as a result of the interaction of the described processes. Beyond that, other phenomena, such as the absorption-induced dislocation emission (AIDE) or hydrogen-enhanced strain-induced vacancy formation (HESIV), can take a part in the crack formation. Nevertheless, in the present study on the AISI 304L steel, these two mechanisms should be of less importance^39^.

For instance Tavares *et al*. claimed that typical features of the decohesion are (quasi-)cleavage and intergranular fracture^40^. This becomes obvious in the micrograph in Fig. 3 where cracking occurs along grain and twin boundaries. Differences in the orientation of neighbouring grains cause a mismatch between them. As a result of that, highest stress and, thus, cracking occurs along grain boundaries with highest misorientation angle. Due to the loss in cohesive energy in the presence of deuterium, the localised stress concentrations are not accommodated plastically. However, the SEM micrograph in Fig. 3 reveals several short transgranular cracks too. These formed mainly parallel to slip bands and at their intersections.

The distribution of deuterium in the matrix is heterogeneous. Areas with a higher intensity of deuterum-related signals (orange) can be found as well as locations with a relatively lower intensity (yellow) and regions where the signal was too weak to be detected (blue). Considering Fig. 1(c) together with Fig. 3(a), it becomes obvious that deuterium is dissolved in both the austenite and trapped by the newly formed martensite. Solute deuterium in the FCC phase after charging can be explained by its high solubility. The presence of deuterium in the BCC phase, however, stands against the common assumption of its low solubility for hydrogen and deuterium, respectively. Martensite contains a high number of defects. Especially dislocations that form during transformation are regarded as effective trapping sites for hydrogen, and in consequence, for deuterium^41^. Kim *et al*. showed furthermore that interfaces, grain boundaries and point defects within the newly formed martensite bind hydrogen^42^. The white arrow in Figs. 1(c) and 3 shows the accumulation of deuterium already at slip lines, i.e. the precursor of martensite. In addition to that, it is anticipated that deuterium may not only be trapped at these defects but also dissolved within the lattice. Due to the rapid *γ*-*α*-transformation, diffusion of deuterium was restricted so that the newly formed BCC matrix was supersaturated with deuterium. It was already discussed in a previous work that deuterium can be incorporated into the newly formed BCC/HCP lattice^20^.

The present observations of deuterium being trapped by martensite support recent findings of Pu *et al*.^43^. The authors investigated the changes in hydrogen desorption resulting from martensitic transformation. In their work, bainitic steels with retained austenite were electrochemically charged, compressed and subsequently analysed by thermal desorption analysis (TDA). A decreased hydrogen effusion from the compressed samples in comparison to mechanically unloaded reference samples was measured. Supplementary X-ray diffraction measurements (XRD) suggested an increase in dislocation density from 5×10^14^ to 4×10^15^, acting as traps for hydrogen.

Results contrasting the present observations were obtained e.g. by Koyama *et al*.^7,44,45^. The authors cooled down hydrogen pre-charged samples from different austenitic steel grades to cryogenic temperatures and determined the evolution of hydrogen. An increased hydrogen release rate during the formation of athermal *ϵ*-martensite was observed. The explanation for this behaviour was the lower solubility and higher diffusivity of hydrogen in martensite in comparison to the parent austenite.

From these findings and the present results, the question arises of how similar solubility and diffusivity for hydrogen of *ϵ*- and *α*’-martensite are. It also has to be clarified in which way the trapping behaviour of strain- and stress-induced martensite differ from athermal martensite.

A thorough and systematic comparison of athermal martensite transformation (AMT) and deformation-induced martensite transformation (DIMT) was done by Tian *et al*.^46^. For this purpose, the authors investigated several austenitic steel grades with nickel concentrations varying from 10.5 to 14 wt.-%. The formation and structure of AMT and DIMT in steels with a low stability of the austenite phase, i.e. low nickel concentration, was indicated by SEM and EBSD observations. From these results it can be anticipated that the currently investigated grade AISI 304L, possessing a relatively low stability of the *γ*-phase, behaves in the same way.

In order to fully exploit the information content of the ToF-SIMS results, principal component analysis (PCA) was applied^47^. This allowed to incorporate not only the deuterium signal but other deuterium-related fragments such as OD^−^. The obtained principal components (PC) contain several peaks of different masses that were detected during SIMS analysis. The influence of each peak within the principal component is weighed which shows the contribution of an element to the PC. Deuterium together with several deuterium-related fragments is reflected by PC2 in the measurement *without* external mechanical load, shown in Fig. 3(a). In contrast to that, PCA on the data gathered *during* applying mechanical loading resulted in the deuterium signals condensed in PC1. The first two to three PCs are typically containing the main amount of variance and are therefore used for data interpretation^48^. The variance of higher PCs is usually very small or even zero. Consequently PC1 explains a higher amount of the total variance than PC2. The deuterium-related peaks had a higher variance after applying the mechanical load. This gives an indication of the stronger signal intensity as a result of the strain.

During mechanical loading of the specimen, the part of the sample farther away from the notch encountered compressive stress, while in the vicinity of the notch tensile stress prevailed. The highest stress and strain occurred directly below the notch. The material was plastically strained in the notch root. The size of this plastic zone was approximately 65 μm. The field of analyses was located about 85 μm from the notch, where the material was deformed elastically. However, this was sufficient to induce a movement of deuterium towards the notch, viz. the area under tensile stress. This was energetically favourable since tensile stress expanded the lattice, offering more space for deuterium^49^. The comparison of Fig. 3(a,b) reveals that not only the overall intensity of the deuterium-related signals is higher in Fig. 3(b) as compared to (a). Additionally, the concentration of deuterium increases towards the upper part of the ROI, thus, towards the notch where the highest stress and strain are assumed to occur.

The sample was cooled down to a temperature of  ≈ −70±5°C during the experiments. This was done to hamper diffusion of deuterium. Although diffusion does occur even at this low temperature, its strong influence as a possible artefact can be reduced here. To corroborate the viability of the PCA applied on the SIMS data, representative masses were selected and analysed. The FeO^−^ and CrO^−^ signals were selected, for iron and chromium are the main alloying elements of the investigated grade AISI 304L. Thus, the selected masses should not be subjected to alterations during mechanical loading. The same applies to the ubiquitously present hydrogen in the analysis chamber. That is why the signal of the fragment OH^−^ was chosen as well. Indeed, the comparison of each mass before and during mechanical loading did not show differences. For this reason it is assumed that scaling of the data and principal component analyses did not bias the results.

Therefore, it can be concluded that the re-distribution of deuterium occurred predominantly due to the strain-induced motion of deuterium as it was reported, e.g., by Lufrano *et al*.^49^. In this work the authors simulated hydrogen diffusion in four-point bend specimen and showed that the increased lattice spacing during bending decreased the chemical potential of hydrogen in solid solution. The resulting gradient of the chemical potential triggered movement of hydrogen towards the crack tip. Yokobori *et al*. confirmed this behaviour by numerical analyses^50,51^. Their model showed that hydrogen diffuses along a stress gradient and accumulates in regions of maximum triaxial tensile stress.

Recently, McMahon *et al*. were able to confirmed this by SIMS experiments^52^. They conducted fatigue tests on steel grade AISI 316 under high-purity deuterium gas atmosphere. The distribution of deuterium in the vicinity of the fatigue crack was then imaged by subsequent NanoSIMS measurements. Dislocation tangles ahead and in the wake of the crack tip, i.e. within its plastic zone, as well as interfaces of MnS inclusions exhibited a higher concentration of deuterium than the surrounding material. From that, the authors concluded that dislocation-mediated transport of deuterium occurred and the role of inclusions as traps for hydrogen was evident.

This so-called *dislocation drag* of deuterium^53^ is of less importance at the low strains shown in the present results. However, the mechanical bending-induced stress field was sufficient to prompt deuterium movement, resulting in an increased signal intensity. Therefore, it can be taken as an indication and proof of the *Gorsky effect*. An additional driving force could furthermore have been the tensile stresses that resulted from contraction of the lattice during deuterium effusion.

## Conclusions

- The austenitic stainless steel AISI 304L was electrochemically charged with deuterium for 120 hours. SEM investigations revealed severe intergranular cracking along grain boundaries as well as shorter transgranular cracks. Both formed due to the high fugacity charging and the resulting internal stresses in the lattice. Mechanisms such as HEDE and shape deformation due to mismatch between the neighbouring grains and sub-grains provided additional driving forces for surface cracking.
- The comparison of the phase distribution maps taken before and after electrochemical charging with D_2_SO_4_ revealed the transformation of the pristine austenite into *ϵ*- and *α*’-martensite in some locations of the surface. Nucleation sites for martensite were slip bands. This is in accordance to the formation sequence of strain-induced martensite described in the literature.
- ToF-SIMS measurements after electrochemical charging exhibited accumulation of deuterium in the austenite as well as in the newly-formed martensite. However, it cannot be conclusively answered whether the HCP/BCC phase was supersaturated with deuterium or deuterium was trapped by defects that evolved during phase transformation.
- A custom-made sample holder for applying external mechanical load onto the specimen *during* ToF-SIMS analysis was successfully tested. Comparison of the deuterium distribution before and during the application of mechanical load revealed a partial re-distribution of deuterium towards the region of the highest stress. It can be assumed that this is due to the Gorsky effect.

## Methods

### Materials and sample preparation

The investigated steel grade AISI 304L was purchased from *ThyssenKrupp Schulte GmbH (Dortmund, Germany)*. It was cold-rolled, annealed, pickled and in a step soft rolled to a final thickness of 0.5 mm by the supplier. Its chemical composition is shown in Table 1.

**Table 1:** Chemical composition of the investigated material according to the supplier. All values are given in wt.-%.

C	N	Cr	Ni	Mn	Si	P	S	Fe
0.022	0.053	18.25	8.05	1.53	0.38	0.033	0.002	bal.

Notched bending specimens were cut out by electric discharge machining (EDM) from the sheet material. This was done to prevent any cold deformation. Subsequently, the surface of the samples was mechanically ground with SiC papers down to 4000 grit and polished with 3 and 1 μm diamond suspension and 0.25 μm colloidal silica for five minutes per step. To fully remove all residuals of martensite introduced by the rolling process and to further increase the surface quality, the ROI below the notch was polished by five seconds electrolytic polishing with the solution A2 from *Struers GmbH (Berlin, Germany)*.

#### Electrochemical charging

The introduction of deuterium was realised by electrochemical charging for 72 hours in a solution of 0.05M D_2_SO_4_. A current density of 5 mA/cm^2^ was applied. During charging, the electrolyte was kept low on oxygen by constantly purging it with high purity gaseous nitrogen.

#### Scanning electron microscopy and electron back-scatter diffraction

Scanning electron microscopy images have been captured in a Supra 40 and a LEO 1530VP *(Garl Zeiss GmbH, Oberkochen, Germany)*. In order to gain insight into the deuterium-induced phase changes, the LEO 1530VP was equipped with a phosphorus screen and an EBSD detector *e*^−^*Flash*^*HR*^*(Bruker Nano, Berlin, Germany)*. The acceleration voltage in all measurements was 20 kV.

#### Time-of-flight secondary ion mass spectrometry

**Figure 4:**
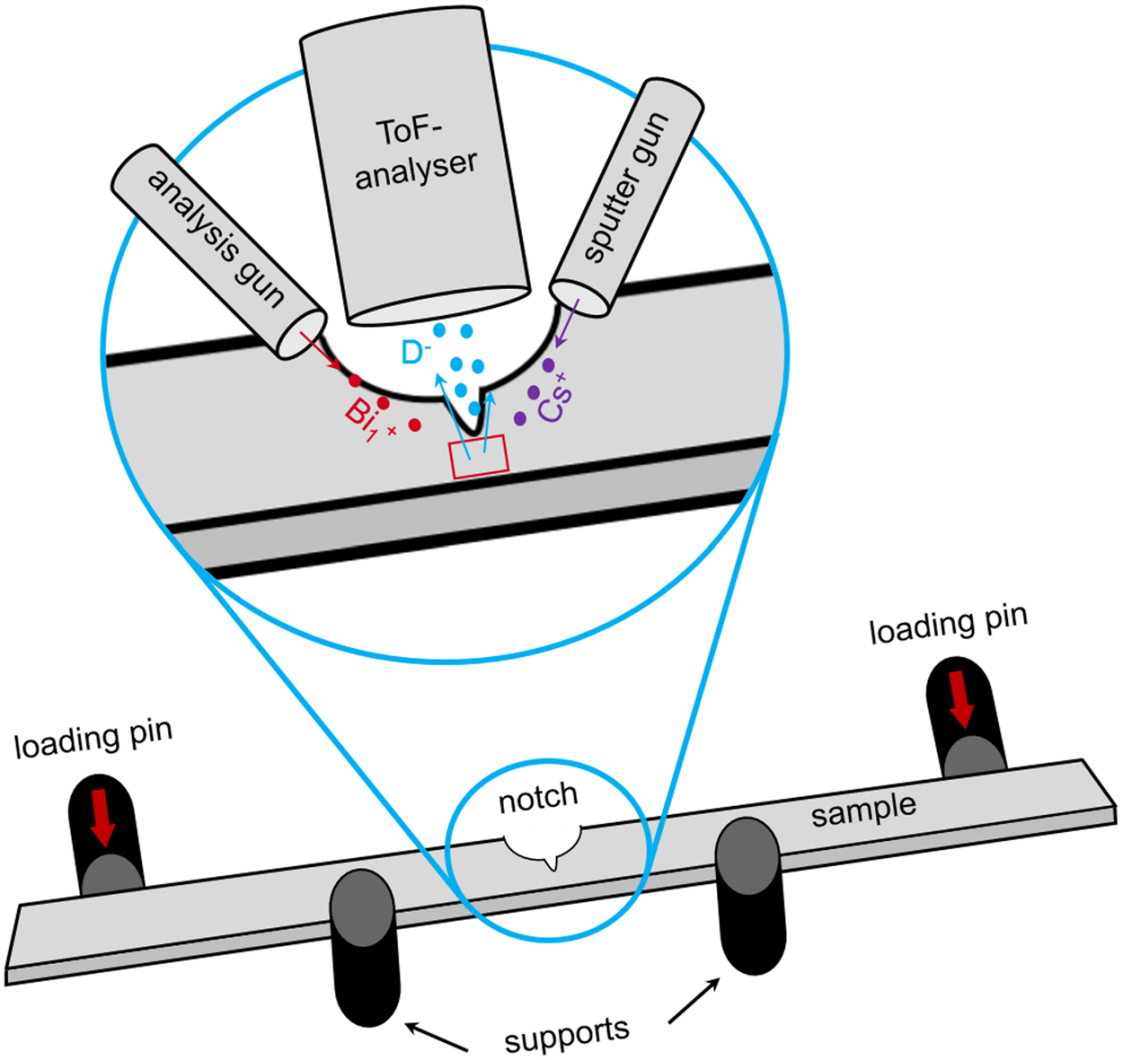
Sketch of the setup of the ToF-SIMS experiments. Secondary ion images were taken both *before* and *during* mechanical loading. Bismuth ions were used for analysis, caesium ions for a preceding sputter step. The analyser gathered only negatively charged secondary ions.

Analyses of the deuterium distribution were done with a TOFSIMS IV *(IONTOF GmbH, Münster, Germany)*. Micro four-point-bending specimens were mechanically ground and polished and electrochemically polished. Subsequently, they were charged. To prevent outgassing of deuterium from the sample, it was cooled to  −70°C ± 5°C. An area of 400×400 μm^2^ was sputtered for five minutes with a 1 keV Cs^+^ beam directly before ToF-SIMS analyses. This was done to remove contaminations and to enhance the secondary ion yield of deuterium^54^. The data was acquired using the collimated burst alignment mode (CBA) using a 25 keV Bi${}_{1}^{+}$ ion beam^55^. A ROI of 150×150 μm^2^ was scanned in a sawtooth mode with 512 by 512 pixels and one shot per pixel. Figure 4 gives a sketch of the experimental setup of the ToF-SIMS.

External mechanical load was applied on the specimen by an innovative, custom-made four-point-bending sample holder. The load transmission was realised by two piezo actuators type PA 50/T14 (“loading pin” in Fig. 4) purchased from *piezosystem jena GmbH (Jena, Germany)*. By that, a displacement of ≤50 μm was accomplished. The first SIMS analysis was performed *without* applying mechanical load, whereas the second (on the same ROI) was performed *while* applying the mechanical load. In the latter case, the sample was constantly strained during the acquisition. The highest mechanical stress developed in the notch root was approximately 365 MPa. A detailed description of the four-point-bending device can be found in^56^.

#### Data treatment

Image drift due to temperature fluctuations and the sample movement by bending required a shift correction of the raw image data. The fusion of SEM and SIMS data as well as the principal component analysis (PCA) were done with the software ImageLab by *Epina Softwareentwicklungs- und Vertriebs-GmbH (Retz, Austria)*. Scaling of the data ahead of the PCA equalised the noise level of all peaks to the same order of magnitude. Thus, variances of the signals became visible and peaks comparable^20^. The PCA was applied on the acquired SIMS image data to consider covariant features, such as deuterium fragments which are bound to oxygen. Applying PCA revealed features like this and hence increased the information content significantly.
